# Supplementary material for: Seminoma and Embryonal Carcinoma Footprints Identified by Analysis of Integrated Genome-Wide Epigenetic and Expression Profiles of Germ Cell Cancer Cell Lines
Source: PLoS One. 2014 Jun 2;9(6):e98330. doi: 10.1371/journal.pone.0098330 (PMC4041891; doi:10.1371/journal.pone.0098330)
Supplement: File S1 — ZIP file containing DMRforPairs output for significant regions. Please start from the html files. (ZIP) [file pone.0098330.s008.zip › figures/8245.pdf]

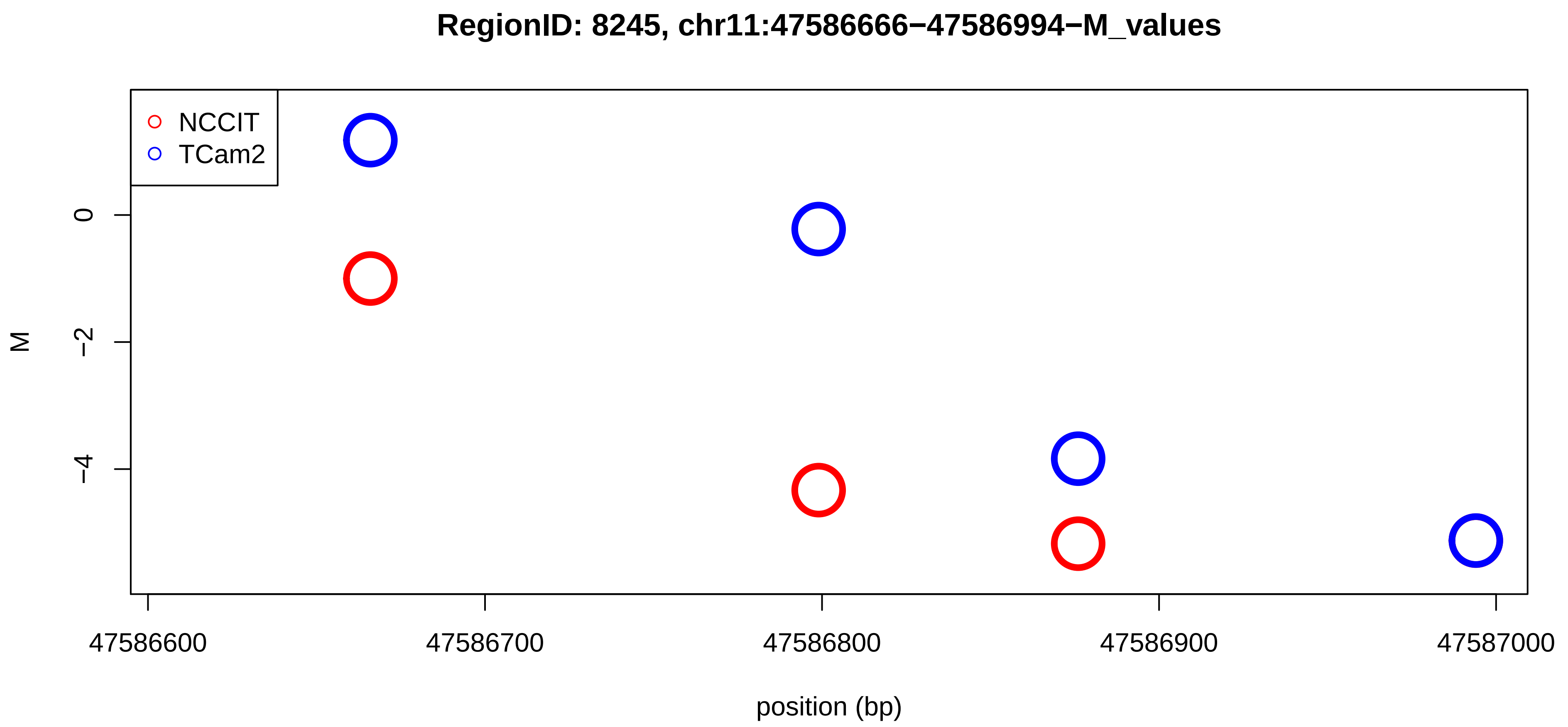
RegionID: 8245, chr11:47586666−47586994−M_values
NCCIT
TCam2
0
−2
−4
M
47586600
47586700
47586800
47586900
47587000
position (bp)

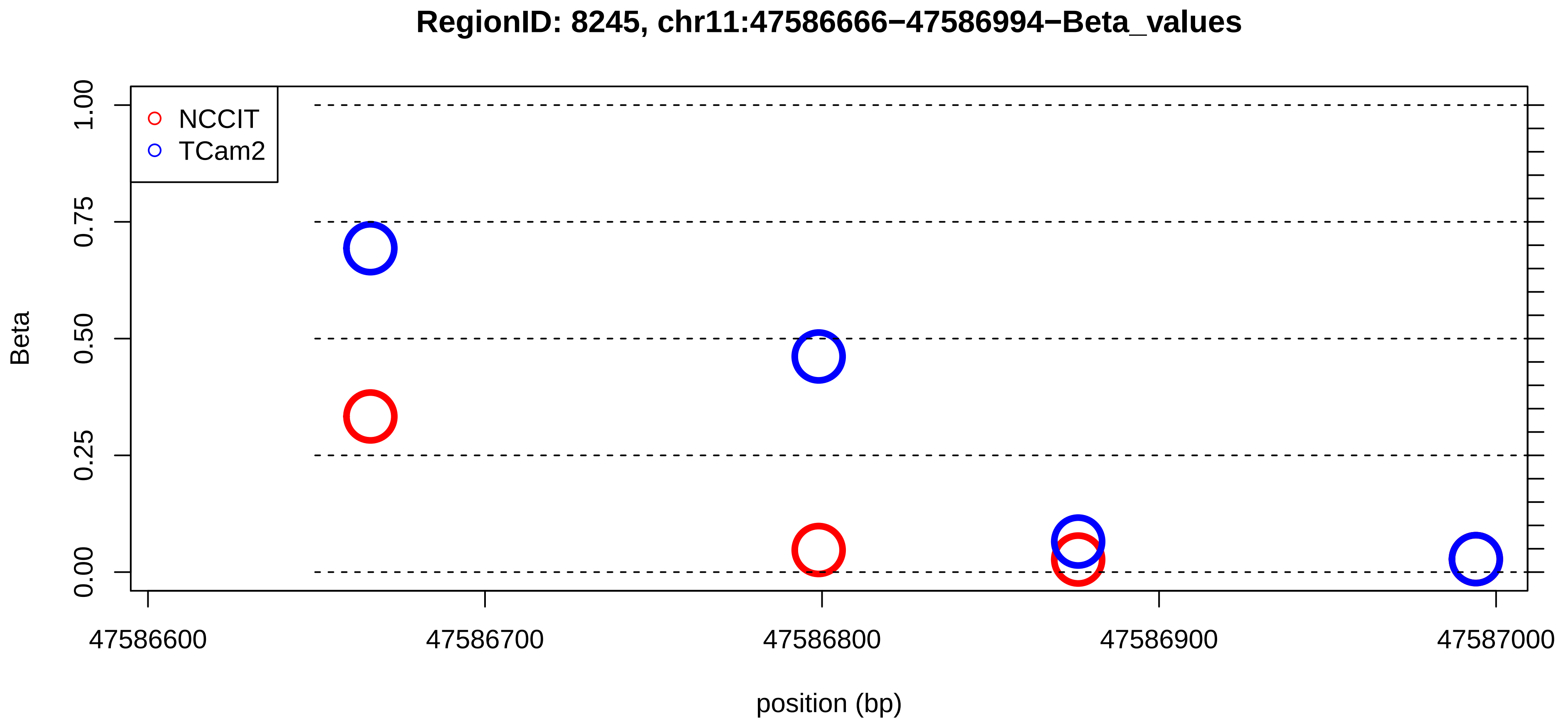
RegionID: 8245, chr11:47586666−47586994−Beta_values
NCCIT
TCam2
1.00
0.75
0.50
0.25
0.00
Beta
47586600
47586700
47586800
47586900
47587000
position (bp)
